# The heterogeneous life space trajectories and predictors in stroke patients: a cohort study

**DOI:** 10.3389/fneur.2025.1627893

**Published:** 2025-10-03

**Authors:** Bei Yang, Rui Xie, Siyuan Ge, Ruixue Tang, Kangyao Cheng, Yin Wang

**Affiliations:** ^1^Shanghai Ninth People’s Hospital, Shanghai Jiao Tong University School of Medicine, Shanghai, China; ^2^School of Nursing, Shanghai University of Traditional Chinese Medicine, Shanghai, China

**Keywords:** life space, stroke, trajectories, predictors, latent class growth model

## Abstract

**Objective:**

This study aimed to identify heterogeneous trajectories of life space among stroke patients and explore the predictors for different classes of life space.

**Methods:**

This prospective cohort study assessed 210 stroke patients’ life space at baseline and 1, 3, 6 months post-discharge. We elucidated heterogeneous trajectories of life space by latent class growth model and explored the predictors of trajectories by multinomial logistic regression analysis.

**Results:**

Among 173 participants completing the 6-month follow-up, three distinct life space trajectories were identified: the “high-level recovery flat class” (8%), the “medium-level recovery good class” (72%), and the “low-level recovery poor class” (20%). Multinomial logistic regression, using the low-level recovery poor class as the reference, indicated that age <60, absence of limb sensory deficit, and positive environmental experiences were predictors of the medium-level recovery good class, whereas employment status and positive environmental experiences were predictors of the high-level recovery flat class.

**Conclusion:**

The three trajectories of life space indicated that the 1 month post-discharge is the most vulnerable phase for stroke patients. Age and employment status significantly influence life space trajectories. Patients in the low-level recovery poor class should receive special attention. Strategies to improve sensory deficits and environmental experiences should be developed to expand life space, promoting stroke patients’ rehabilitation.

## 1 Introduction

Stroke is defined as an injury to brain tissue resulting from a sudden blockage or rupture of an artery that supplies blood to the brain (1). In 2021, there were 93.8 million stroke survivors globally, ranking stroke as the third leading cause of death and a leading cause of disability (2). In China, stroke represents the foremost cause of death and disability among adults, with over 26.3 million prevalent cases and a significant annual incidence, resulting in the highest stroke-related disease burden globally (2–4).

Following a stroke, approximately 70 to 80% of patients experience varying degrees of physical impairments, like hemiparesis, muscle weakness, and aphasia (5). These impairments not only limit patients’ mobility but also contribute to various psychosocial issues, including depression, loneliness, and social isolation (6, 7). Clearly, stroke survivors encounter health challenges across physiological, psychological, and social domains, attributable to the disease and its sequelae, significantly impacting their quality of life and increasing the risk of recurrence. The progression of stroke is stage-specific, with patients showing different changes in physical function and psychosocial status at different stages. Previous studies have indicated that the 1, 3, and 6 months post-stroke are critical periods for changes in physiological functions and psychosocial status of patients (8, 9). Therefore, developing a comprehensive and dynamic monitoring indicator with high sensitivity is crucial for effective patient health assessment and follow-up.

Life space, defined as the spatial extent encompassing an individual’s daily activities, reflects habitual activity performance in daily life (10). The extent of the life space is modulated by multiple factors, including physical, cognitive, psychological, and social environmental determinants (11). Therefore, life space can be seen as a comprehensive representation of an individual’s physical activity capacity, psychological and social functioning in relation to environmental demands. It constitutes a clinically significant metric for evaluating comprehensive recovery outcomes in stroke patients. Moreover, life space is inherently dynamic, and significant variations can be observed across different stages of stroke (12, 13). Trajectories showing significant improvement in life space are often indicative of good recovery, while persistently restricted life space may signify poor functional outcomes and a higher need for clinical intervention (14, 15).

However, few studies have systematically investigated the longitudinal changes in life space among stroke patients. Therefore, the study of the life space trajectories and the description of population characteristics deserve further investigation. To address this research gap, two methodological challenges must be resolved: the application of a feasible statistical approach to classify life space trajectories and the identification of predictors associated with each trajectory. Previous longitudinal studies have predominantly employed aggregated variable analyses to evaluate overall trajectories, which often overlooks the heterogeneity among individual participants (12). In this context, latent class growth model (LCGM) analysis enables the identification of individual differences and determines categories or subgroups with similar trajectory patterns concerning the target dependent variable (16). This data analysis method provides a more nuanced view on the way life space changes over time by modeling individual life space patterns, thus providing the opportunity to explore the association with life space and any survey point within trajectory class (17). Consequently, this study aimed to: (1) characterize the trends in life space changes within 6 months post-discharge for stroke patients; (2) identify the heterogeneous trajectories of life space among stroke patients; and (3) explore the predictors of life space changes over time in patients with different trajectories.

## 2 Materials and methods

### 2.1 Study design

This multi-center prospective cohort study was conducted in four Grade III Level A hospitals—the highest rank in the Chinese hospital accreditation system, denoting large-scale, tertiary-care referral centers with the highest standards of medical expertise and equipment—from February 2023 to August 2023 in Shanghai, China. Stroke patients completed the self-deigned questionnaire, along with the scales of life space, physical functioning, self-efficacy, hope, psychological distress, social support, economic status and built environment at baseline (within 1 week post-discharge, T0). Participants were additionally required to complete three consecutive follow-up assessments of life space at 1 (T1), 3 (T2), and 6 (T3) months post-discharge. Ethical approval regarding human subject research was obtained from the Ethics Committee on Shanghai University of Traditional Chinese Medicine (approval number: 2023-1-13-08), and each participant provided informed consent.

### 2.2 Participants

The inclusion criteria were as follows: (1) were infarction or hemorrhagic stroke patients who were at least 18 years old. All stroke diagnoses were confirmed by brain magnetic resonance imaging or computed tomography; (2) were admitted due to acute stroke, and their condition have now stabilized after treatment; (3) stroke severity is mild or greater, specifically the admission National Institute of Health Stroke Scale (NIHSS) scores are ≥2; (4) possessed the ability to write or speak to complete the survey. The exclusion criteria were as follows: (1) with cognitive and or neurological disorders that could interfere significantly with the completion of questionnaires; (2) with mobility-impairing conditions such as visual impairment, lower limb fractures. The total sample size was calculated using the sample size estimation table for single-group repeated measures. According to four repeated measures of life space for each participants, with mean correlation coefficient (*ρ*) = 0.50, *f* = 0.14 (weak effect), *α* = 0.05, (1 − *β*) = 0.8, 142 participants was needed. Considering a dropout rate of 20%, a minimum of 178 participants would be required.

### 2.3 Data collection

Data were collected by trained research team members through a combination of onsite and telephone-based surveys. At baseline (within 1 week post-discharge), eligible participants were approached during their hospital stay. After providing informed consent, participants completed the self-deigned questionnaire for individual characteristics as well as the life space assessment, while other scales, particularly the Measure of Stroke Environment (MOSE), which is sensitive to post-discharge community experience, were administered via telephone after discharge to ensure ecological validity. Follow-up assessments at 1, 3, and 6 months post-discharge were conducted either onsite during scheduled outpatient visits or by telephone for those unable to attend in person. Telephone surveys were performed during predefined time windows (9.00–10.30 a.m. and 2.30–4.00 p.m.) to maximize participant convenience and response rates. Throughout all stages, interviewers could clarify items but were instructed not to influence responses.

### 2.4 Measurements

Adopting the conical model as theoretical guidance, this study identified six predictive domains for life space trajectories (individual characteristics, physical function, psychosocial factors, environment, economy, cognition) (18, 19). As life space assessment required 4-week activity recall, cognitive impairment exclusion criteria established cognition as a control variable.

#### 2.4.1 Life space

Life space was assessed by the Life Space Assessment (LSA), which is a self-report tool measuring the extent of an individual’s actual life mobility, categorized into five hierarchically structured life space areas (outside bedroom, outside home, neighborhood, town, and beyond town) (20). It also evaluates the frequency of mobility within each area (<1 time/week, 1–3 times/week, 4–6 times/week, every day) and the level of independence (personal assistance, device only, no assistance) over the preceding 4 weeks. Based on the life space level, the frequency of reaching each level, and the degree of independence in achieving each level, the scores for each level and the LSA total scores are calculated, ranging from 0 point to 120 points. Higher LSA scores indicate greater life space mobility, while scores below 60 are considered indicative of restricted life space. LSA has been reported to be highly reliable, valid and sensitive to change (21, 22). The Cronbach’s *α* coefficient in this study was 0.745.

#### 2.4.2 Individual characteristics

This study selected the self-designed questionnaire for baseline characteristics and it included two parts: (1) Sociodemographic characteristics such as sex, age, employment status. (2) Disease-related characteristics consisted of National Institute of Health Stroke Scale (NIHSS), type of stroke, presence of dysfunction, comorbidities, etc. (3) Daily going-out habits: the pre-stroke daily going-out habits were assessed using the first subscale of the Chinese version of the Housebound Scale—an instrument that captures whether a participant is housebound or not—and this specific subscale has been verified to possess good construct validity and a reliability coefficient of *α* = 0.74 (23). It evaluates four dimensions over the preceding month: (1) the frequency of spending entire days at home, (2) the frequency of outings for essential activities (e.g., shopping, walks), (3) the frequency of social interactions with friends or relatives, and (4) the usual need for assistance when going out.

#### 2.4.3 Physical function

The physical function of participants was assessed using the Chinese Stroke Scale (CSS), which encompasses myodynamia, speech disorder, mental disorder, walking ability, hemiplegia, and eyeball disorder (24). This scale adopts a scoring range of 0 to 45, where higher scores correspond to more severe neurological deficits; accordingly, neurological impairment severity is classified into three grades based on the scoring criteria: mild (0–15), moderate (16–30), and severe (31–45). The CSS has been validated to exhibit good construct validity and reliability, with a Cronbach’s alpha coefficient of *α* > 0.80 (25).

#### 2.4.4 Psychosocial features

Self-efficacy, hope and psychological distress were selected as the psychosocial features. (1) The Self-Efficacy for Managing Chronic Disease (SEMCD) was used to evaluate perceived adaptability in managing different facets of chronic diseases. Scores range from 6 to 60, with higher scores indicating better self-efficacy (26). The Chinese version was formulated and authenticated with a Cronbach’s alpha of 0.934 (27). In this study, internal consistency of SEMCD was *α* = 0.802. (2) Hope was assessed using the 12-item Herth Hope Index (HHI). Scores range from 12 to 48, with higher scores indicating higher hope (28). The Chinese version was confirmed with a Cronbach’s alpha of 0.85 (29). In this study, internal consistency was *α* = 0.782. (3) Psychological distress was measured with the Kessler Psychological Distress Scale (Kessler 10), which has a scoring range of 0–50 (higher scores reflect more severe psychological distress). Scores are categorized as follows: no distress (10–15), mild distress (16–21), moderate distress (22–29), and severe distress (30–50) (30). The Kessler 10 has been translated into Chinese, with a Cronbach’s alpha of 0.93 (31). In this study, internal consistency was *α* = 0.796.

#### 2.4.5 Environment

This study selected social support and environment assessment as the environment variables. ① The Social Support Rating Scale (SSRS) was used to assess the level of social support, representing the social environment. The SSRS has a scoring range of 12–66, where higher scores indicate better social support. Scores are categorized as low (12–22), moderate (23–44), or high (45–66); the scale also demonstrates good construct validity and reliability (*α* = 0.92) (32). (2) The Measure of Stroke Environment (MOSE) was adopted to evaluate patients’ experience of their built environment, with higher scores indicating that participants have a more positive experience of their built environment. Scales range from 0 to 36, with higher scores indicating that participants have a more positive experience of their built environment (33). The Chinese version of MOSE has excellent internal consistency (Cronbach’s *α* = 0.945, split-half reliability = 0.778) and good convergent validity (34). In this study, Cronbach’s *α* coefficient of the scale was 0.830.

#### 2.4.6 Economy

The Comprehensive Scores for Financial Toxicity Based on the Patient-Reported Outcome Measures (COST-PROM) were utilized to evaluate financial strain. Scales range from 0 to 44, with lower scores indicating heavier economic burden (35). The Chinese version of the COST-PROM exhibits good psychometric properties, with acceptable construct and content validity (36). In this study, the scale’s Cronbach’s *α* coefficient was 0.895.

### 2.5 Statistical analysis

All data were performed using SPSS 25.0 and Mplus 8.3. Descriptive statistics are reported as mean, standard deviation (SD), median, interquartile range (IQR), number and percentage. Participants with missing life space data were excluded. Baseline characteristics were compared using *t*-tests and chi-square tests. LGCM was tested to explore overall trends in life space, with model fit assessed using indices such as the comparative fit index (CFI), Tucker-Lewis index (TLI), root mean square error of approximation (RMSEA), and standardized root mean square residual (SRMR). LCGM was employed to identify subgroups with similar life space trajectories. Five class models were estimated, and the optimal model was determined using the following criteria: (1) lower values of Akaike information criterion (AIC), Bayesian information criterion (BIC), and sample-size-adjusted BIC (aBIC) (37); (2) entropy >0.80, indicating high classification accuracy (38); (3) significant *p* < 0.05 for the Lo–Mendell–Rubin adjusted likelihood ratio test (LMR) and bootstrapped likelihood ratio test (BLRT), suggesting better fit for the k-class model compared to the (*k* − 1) class model (39). Finally, multinomial logistic regression was performed using baseline variables as predictors and the optimal LCGM classes as the dependent variable to identify predictors of trajectory subgroups.

## 3 Results

### 3.1 Participant characteristics

**Figure 1:**
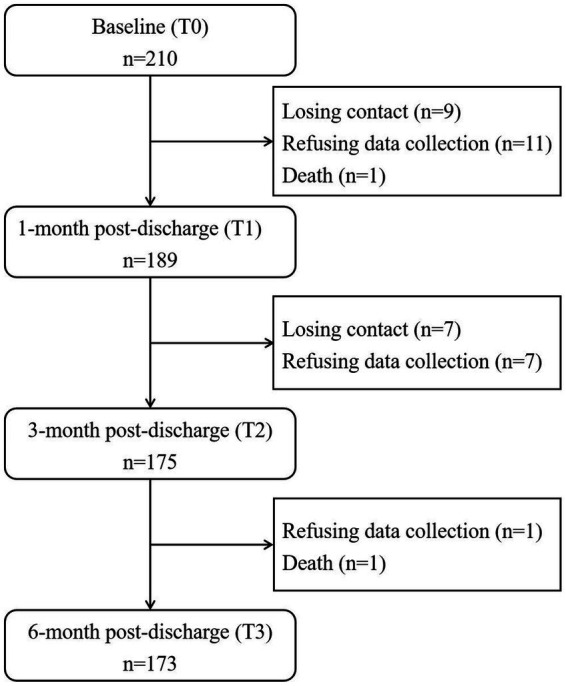
Flowchart of the data collection of the study participants.

Table 1 shows the primary baseline characteristics of the participants (detailed information was shown in Supplementary Table S1). This study recruited 210 stroke patients. Among them, 37 (17.6%) had fewer than three available life space measures from T0 to T3 and were thus excluded from data analysis. Full data were collected from 173 participants. No significant differences in baseline characteristics were observed between the participants who completed three life space assessments and those who were lost to follow-up, indicating that the data were missing at random. Figure 1 presents a flowchart illustrating the participant recruitment process and participant retention rates.

**Table 1:** Baseline characteristics of the study population.

Characteristic		*n* (%)			*p* ^a^	*p* ^b^
		Overall (*n* = 210)	Completed three follow-ups (*n* = 173)	Lost to follow-up (*n* = 37)		
Age (years)	<60	83 (39.5)	68 (39.3)	15 (40.5)	0.965	0.889
	≥60	127 (60.5)	105 (60.7)	22 (59.5)		
Sex	Male	150 (71.4)	128 (74.0)	22 (59.5)	0.559	0.076
	Female	60 (28.6)	45 (26.0)	15 (40.5)		
Place of residence	Urban	165 (78.6)	136 (78.6)	29 (78.4)	0.695	0.146
	Town	43 (20.5)	37 (21.4)	6 (16.2)		
	Rural	2 (0.9)	0 (0)	2 (5.4)		
Employment status	Employed	19 (9.0)	15 (8.7)	4 (10.8)	0.997	0.917
	Employed (be on sick leave)	49 (23.3)	41 (23.7)	8 (21.6)		
	Unemployed	22 (10.5)	19 (11.0)	3 (8.1)		
	Retired	120 (57.1)	98 (56.6)	22 (59.5)		
Family income, thousand/m (RMB)	1,000–3,000	2 (1.0)	1 (0.6)	1 (2.7)	0.946	0.380
	3,001–5,000	31 (14.8)	27 (15.6)	4 (10.8)		
	5,001–10,000	116 (55.2)	98 (56.6)	18 (48.6)		
	>10,000	61 (29.0)	47 (27.2)	14 (37.8)		
Type of stroke	Ischemic stroke	193 (91.9)	157 (90.8)	36 (97.3)	0.689	0.321
	Hemorrhagic stroke	17 (8.1)	16 (9.2)	1 (2.7)		
No. of stroke occurrences	1	155 (73.8)	129 (74.6)	26 (70.3)	0.934	0.481
	2	44 (21.0)	34 (19.7)	10 (27.0)		
	≥3	11 (5.2)	10 (5.8)	1 (2.7)		
No. of other chronic diseases	0	41 (19.5)	35 (20.2)	6 (16.2)	0.980	0.804
	1–2	144 (68.6)	117 (67.6)	27 (73.0)		
	≥3	25 (11.9)	21 (12.1)	4 (10.8)		
No. of functional impairments	0	38 (18.1)	34 (19.7)	4 (10.8)	0.924	0.429
	1–2	127 (60.5)	102 (59.0)	25 (67.6)		
	≥3	45 (21.4)	37 (21.4)	8 (21.6)		
Participation in social activities	Yes	105 (50)	83 (48.0)	22 (59.5)	0.693	0.205
	No	105 (50)	90 (52.0)	15 (40.5)		

No., number.

a Comparison of characteristics between a total of 210 participants and 173 participants who completed three follow-ups.

b Comparison of characteristics between 173 participants who completed three follow-ups and 37 participants who were lost to follow-up.

### 3.2 Changes in life space of stroke patients at different stages

**Figure 2:**
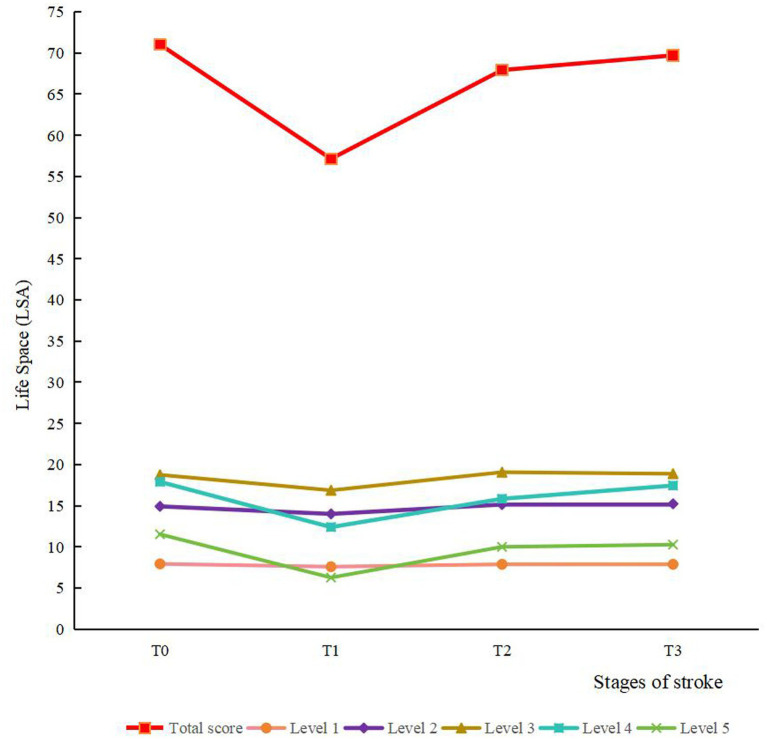
The trends of scores for life space and various levels. Level 1, outside bedroom; level 2, outside home; level 3, to neighborhood; level 4, to town; level 5, beyond town.

The results of the descriptive analysis of life space measured from T0 to T3 are shown in Table 2 and Figure 2. The average total life space scores at the four time points were 71.03, 57.14, 67.93 and 69.71, respectively. The scores of the five levels of life space also showed a falling and then rising trend from T0 to T3.

**Table 2:** Characteristics of life space from T0 to T3 (*n* = 173).

Variable (mean ± SD)	T0 (within 1 week post-discharge)	T1 (1-month post-discharge)	T2 (3-month post-discharge)	T3 (6-month post-discharge)
Life space	71.03 ± 19.15	57.14 ± 22.14	67.93 ± 20.00	69.71 ± 19.97
Level 1^a^	7.93 ± 0.43	7.59 ± 1.05	7.88 ± 0.64	7.88 ± 0.71
Level 2^b^	14.92 ± 2.39	14.00 ± 3.36	15.14 ± 2.29	15.23 ± 2.11
Level 3^c^	18.75 ± 5.08	16.87 ± 6.39	19.07 ± 5.44	18.88 ± 5.82
Level 4^d^	17.90 ± 9.51	12.40 ± 9.89	15.84 ± 9.40	17.45 ± 9.08
Level 5^e^	11.53 ± 11.08	6.27 ± 7.89	10.00 ± 9.80	10.27 ± 9.63

SD, standard deviation.

a Level 1: outside bedroom.

b Level 2: outside home.

c Level 3: to neighborhood.

d Level 4: to town.

e Level 5: beyond town.

The scoring ranges are as follows: level 1 (0–8), level 2 (0–16), level 3 (0–24), level 4 (0–32), and level 5 (0–40), with the total LSA scores ranging from 0 to 120.

Table 3 shows the results of the ANOVA on the scores from T0 to T3 of the total life space and its five levels. The scores of life space and its five levels were the lowest at T1, which indicated that the first month post-discharge was the most traumatized period of the patient, while the third month post-discharge showed significant improvement in patients’ life space and a notable recovery of all functions. Additionally, at level 4, the T3 score exceeded the T2 score. This shows that patients’ activity levels in their local town area decreased initially and then continuously increased in the 6 months post-discharge.

**Table 3:** Repeated measures ANOVA on the scores of life space and various levels of T0–T3.

Variable	*F*	*p*	Multiple comparisons (*p* < 0.05)
Life space	39.690	<0.001	T0 > T1, T2 > T1, T3 > T1
Outside bedroom	13.423	<0.001	T0 > T1, T2 > T1, T3 > T1
Outside home	12.301	<0.001	T0 > T1, T2 > T1, T3 > T1
Neighborhood	10.464	<0.001	T0 > T1, T2 > T1, T3 > T1
Town	20.847	<0.001	T3 > T2 > T1, T0 > T1
Beyond town	18.981	<0.001	T0 > T1, T2 > T1, T3 > T1

### 3.3 Descriptive statistics of other variables

Based on the conical model of life space, we investigated the following seven variables: physical function, self-efficacy, hope, psychological distress, social support, economic status and built environment at baseline. The characteristics of these variables of are presented in Table 4.

**Table 4:** Characteristics of other variables at baseline (*n* = 173).

Variable	Min	Max	Mean ± SD/M (P_25_, P_75_)
CSS score	0	30	5 (2, 9.5)
SEMCD score	16	60	38.81 ± 8.47
Disease management	10	40	25.82 ± 6.37
Health behavior	4	20	12.99 ± 3.41
HHI score	15	45	35.79 ± 4.04
Temporality and future	6	15	11.14 ± 1.54
Positive readiness and expectancy	4	16	11.92 ± 1.42
Interconnectedness	5	16	12.73 ± 1.91
Kessler 10 score	10	39	14 (12, 18)
SSRS score	20	57	36.48 ± 6.19
Subjective support	11	32	21.04 ± 4.06
Objective support	3	14	8.54 ± 1.90
Support utilization	3	12	6.90 ± 2.16
COST-PROM score	5	42	31 (26, 36)
MOSE score	7	36	27 (24, 29)

SD, standard deviation; M, median; CSS, Chinese Stroke Scale; SEMCD, Self-Efficacy for Managing Chronic Disease; HHI, Herth Hope Index; Kessler 10, Kessler Psychological Distress Scale; SSRS, Social Support Rating Scale; COST-PROM, Comprehensive Scores for Financial Toxicity Based on the Patient-Reported Outcome Measures; MOSE, Measure of Stroke Environment.

### 3.4 Latent growth curve model

The LGCM was constructed from the life space data collected from T0 to T3, and the results showed that the unconditional nonlinear model fitted the best (Supplementary Table S2). It indicated that there was significant variability in both the patients’ initial level of life space and the average growth rate, which represents the existence of group heterogeneity in the trajectory of life space change, and also suggests that further exploration of heterogeneous trajectories of life space in stroke patients is warranted.

### 3.5 Latent class growth model

**Figure 3:**
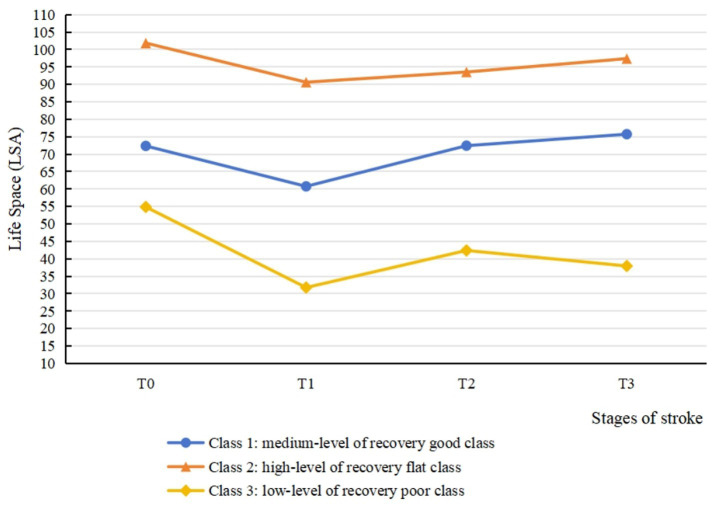
Three-class latent class growth model for life space from baseline to 6 months post-discharge.

The model fitting results for latent classes 1 to 5 using LCGM is summarized in Table 5. In the Class 3 model, the values of AIC, BIC, and aBIC were smaller than in the Class 1 and Class 2. The *p* values of LMR and BLRT test were significant in the Class 3 model (*p* < 0.05) but not in the Class 4 and Class 5 models. Moreover, the information entropy in the Class 3 model was >0.8. Considering the values of fitting indicators and the practical significance of potential categories, it was appropriate to divide the heterogeneous trajectory of life space into three classes. Figure 3 shows the result of the class-specific trajectory of life space. Based on their distinct patterns of change over time and the clinical implications inferred from their life space levels, the three trajectories were labeled as follows: ① medium-level of recovery good class (Class 1, 72%): this group experienced an initial decline in life space but subsequently demonstrated a significant positive slope, ultimately achieving life space levels that exceeded their baseline measurement by the 6-month follow-up. This robust and sustained upward trajectory is indicative of a positive functional recovery process. ② High-level of recovery flat class (Class 2, 8%): this group maintained a consistently high life space level from baseline, indicating minimal disruption and successful preservation of pre-stroke mobility function. ③ Low-level of recovery poor class (Class 3, 20%): this group exhibited a low initial score coupled with a markedly attenuated slope of improvement. Their life space remained persistently restricted throughout the study period, reflecting a high level of unmet rehabilitation needs and poor functional recovery.

**Table 5:** Five models fitting effects (*n* = 173).

Class	*k* ^a^	AIC	BIC	aBIC	Entropy	LMR	BLRT	Number of people in the class (class probability)
1c	8	5999.922	6025.149	5999.816	—	—	—	173 (1.00)
2c	12	5911.747	5949.586	5911.587	0.841	0.0365	<0.001	136 (0.79)/37 (0.21)
3c	16	5888.286	5938.738	5888.073	0.861	0.0123	<0.001	125 (0.72)/13 (0.08)/35 (0.20)
4c	20	5871.094	5934.160	5870.829	0.838	0.4787	<0.001	12 (0.07)/112 (0.65)/15 (0.08)/34 (0.20)
5c	24	5855.437	5931.116	5855.119	0.872	0.2348	<0.001	5 (0.03)/7 (0.04)/34 (0.20)/114 (0.66)/13 (0.07)

AIC, Akaike information criterion; BIC, Bayesian information criterion; aBIC, sample-size-adjusted BIC; LMR, Lo–Mendell–Rubin adjusted likelihood ratio test; BLRT, bootstrapped likelihood ratio test.

a Number of free estimated parameters.

### 3.6 Predictors of life space trajectories

The primary results of univariate analysis between different classes are presented in Table 6 (detailed information was shown in Supplementary Table S3). Variables with *p* < 0.05 in univariate analyses were selected into the multinomial logistic regression model to identify the predictors of the life space trajectories. The overall model fit was statistically significant (likelihood ratio test: *χ*^2^ = 104.532, *p* < 0.001). Nagelkerke pseudo *R*^2^ = 0.583, indicating strong explanatory power of the model. The results, including odds ratios (OR) and their 95% confidence intervals (CI) as measures of effect size and precision, are presented in Table 7. Compared with patients in Class 3, patients younger than 60 years old (OR = 7.404, 95% CI: 1.440–38.079), without sensory impairment (OR = 3.043, 95% CI: 1.015–9.121), and with better environmental experience (OR = 1.244, 95% CI: 1.088–1.424) were more likely to be Class 1. Moreover, employment status (OR = 29.621, 95% CI: 1.186–739.708), and with better environmental experience (OR = 1.999, 95% CI: 1.363–2.931) were associated with greater odds of belonging to Class 2 than Class 3.

**Table 6:** Univariate analysis of life space trajectories.

Variable		Class 1 (*n* = 125)	Class 2 (*n* = 13)	Class 3 (*n* = 35)	*F*/*H*/*χ*^2^	*p*
Age (years), *n* (%)	<60	52 (41.6)	10 (76.9)	6 (17.1)	15.193^a^	0.001
	≥60	73 (58.4)	3 (23.1)	29 (82.9)		
Sex, *n* (%)	Male	96 (76.8)	12 (92.3)	20 (57.1)	7.941^a^	0.019
	Female	29 (23.2)	1 (7.7)	15 (42.9)		
NIHSS score on admission, *n* (%)	Mild (2 ≤ NIHSS ≤ 4)	92 (73.6)	9 (69.2)	18 (51.4)	6.065^a^	0.048
	Moderate and severe (NIHSS ≥5)	33 (26.4)	4 (30.8)	17 (48.6)		
Place of residence, *n* (%)	Urban	96 (76.8)	11 (84.6)	29 (82.9)	2.062^a^	0.834
	Rural or town	29 (23.3)	2 (15.4)	6 (17.1)		
Employment status, *n* (%)	Employed	42 (33.6)	11 (84.6)	3 (8.6)	25.350^a^	<0.001
	Unemployed	83 (66.4)	2 (15.4)	32 (91.4)		
Family income, thousand/m (RMB), *n* (%)	≤5,000	21 (16.8)	0 (0)	7 (20.0)	8.229^b^	0.072
	5,001–10,000	74 (59.2)	5 (38.5)	19 (54.3)		
	>10,000	30 (24.0)	8 (61.5)	9 (25.7)		
Type of stroke, *n* (%)	Ischemic stroke	115 (92.0)	13 (100)	29 (82.9)	3.397^b^	0.130
	Hemorrhagic stroke	10 (8.0)	0 (0)	6 (17.1)		
Whether first stroke, *n* (%)	Yes	98 (78.4)	11 (84.6)	20 (57.1)	7.263^a^	0.026
	No	27 (21.6)	2 (15.4)	15 (42.9)		
No. of stroke occurrences, *n* (%)	1	98 (78.4)	11 (84.6)	20 (57.1)	8.049^b^	0.064
	2	22 (72.6)	2 (15.4)	10 (28.6)		
	≥3	5 (4.0)	0 (0)	5 (14.3)		
No. of other chronic diseases, *n* (%)	0	24 (19.2)	5 (38.5)	6 (17.1)	5.440^a^	0.220
	1–2	87 (69.6)	8 (61.5)	22 (62.9)		
	≥3	14 (11.2)	0 (0)	7 (20.0)		
No. of functional impairments, *n* (%)	<3	100 (80.0)	10 (76.9)	26 (74.3)	0.555^a^	0.758
	≥3	25 (20.0)	3 (23.1)	9 (25.7)		
Presence of limb movement disorder, *n* (%)	Yes	74 (59.2)	4 (30.8)	26 (74.3)	7.644^a^	0.022
	No	51 (40.8)	9 (69.2)	9 (25.7)		
Presence of limb sensory impairment, *n* (%)	Yes	49 (39.2)	8 (61.5)	22 (62.9)	7.595^a^	0.022
	No	76 (60.8)	5 (38.5)	13 (37.1)		
Presence of visual field defect, *n* (%)	Yes	5 (4.0)	0 (0)	2 (5.7)	0.553^b^	0.798
	No	120 (96.0)	13 (100)	33 (94.3)		
Presence of visual impairment, *n* (%)	Yes	12 (9.6)	2 (15.4)	5 (14.3)	1.364^b^	0.644
	No	113 (90.4)	11 (84.6)	30 (85.7)		
Presence of ataxia, *n* (%)	Yes	9 (7.2)	1 (7.7)	4 (11.4)	0.977^b^	0.638
	No	116 (92.8)	12 (92.3)	31 (88.6)		
Presence of headaches or dizziness, *n* (%)	Yes	18 (14.4)	2 (15.4)	7 (20.0)	0.652^a^	0.722
	No	107 (85.6)	11 (84.6)	28 (80.0)		
Whether to participate in social life, *n* (%)	Yes	62 (49.6)	7 (53.8)	14 (40.0)	1.204^a^	0.548
	No	63 (50.4)	6 (46.2)	21 (60.0)		
Neurological deficit, *n* (%)	Mild	110 (88.0)	12 (92.3)	24 (68.6)	8.505^a^	0.014
	Moderate and severe	15 (12.0)	1 (7.7)	11 (31.4)		
Self-efficacy, *n* (%)	Low level	112 (89.6)	11 (84.6)	33 (94.3)	1.168^a^	0.558
	Medium level	13 (10.4)	2 (15.4)	2 (5.7)		
Hope, *n* (%)	Lower-middle level	53 (42.4)	5 (38.5)	20 (57.1)	7.651^a^	0.087
	High level	72 (57.6)	8 (61.5)	15 (42.9)		
Psychological distress, *n* (%)	No distress	83 (66.4)	9 (69.2)	16 (45.7)	7.926^a^	0.079
	Mild	31 (24.8)	3 (23.1)	10 (28.6)		
	Moderate and severe	11 (8.8)	1 (7.7)	9 (25.7)		
Social support, *n* (%)	Low level	2 (1.6)	0 (0)	2 (5.7)	6.758^b^	0.100
	Medium level	112 (89.6)	11 (84.6)	33 (94.3)		
	High level	11 (8.8)	2 (15.4)	0 (0)		
The score of Housebound Scale, mean ± SD		6.15 ± 1.44	5.77 ± 1.24	6.97 ± 2.22	4.248^c^	0.016
Economic status, M (P_25_, P_75_)		31 (26, 34)	36 (27, 37.5)	32 (20, 36)	5.294^d^	0.071
The score of MOSE, M (P_25_, P_75_)		27 (25, 29)	30 (29, 31)	22 (14, 25)	41.330^d^	<0.001

Class 1, medium-level recovery good class; Class 2, high-level recovery flat class; Class 3, low-level recovery poor class.

NIHSS, National Institute of Health Stroke Scale; M, median.

a *χ*^2^ test.

b Fisher’s exact test.

c ANOVA.

d Nonparametric tests.

**Table 7:** Predictors for trajectory class by multinomial logistic regressio*n* (*n* = 173).

Class	Predictors	*B*	Wald	*p*	OR (95% CI)^a^
Class 1					
	Age <60	2.002	5.741	0.017	7.404 (1.440, 38.079)
	Male	0.448	0.661	0.416	1.565 (0.532, 4.605)
	2 ≤ Admission NIHSS score ≤ 4	0.205	0.103	0.748	1.227 (0.352, 4.281)
	First stroke	0.828	2.278	0.131	2.289 (0.781, 6.706)
	No limb movement disorders	−0.688	1.226	0.268	0.503 (6.706, 1.699)
	No limb sensory deficits	1.113	3.946	0.047	3.043 (1.015, 9.121)
	Mild neurological deficit	0.871	1.195	0.274	2.388 (0.501, 11.375)
	Employed	0.889	1.282	0.258	2.432 (0.522, 11.331)
	The score of MOSE	0.219	10.154	0.001	1.244 (1.088, 1.424)
	The score of Housebound Scale	−0.196	1.260	0.262	0.822 (0.584, 1.157)
Class 2					
	Age <60	1.512	1.195	0.274	4.534 (0.302, 68.166)
	Male	1.012	0.496	0.481	2.750 (0.165, 45.921)
	2 ≤ Admission NIHSS score ≤ 4	−0.378	0.126	0.722	0.685 (0.085, 5.518)
	First stroke	0.427	0.156	0.693	1.532 (0.184, 12.760)
	No limb movement disorders	0.437	0.159	0.690	1.549 (0.180, 13.331)
	No limb sensory deficits	−0.435	0.197	0.657	0.647 (0.095, 4.413)
	Mild neurological deficit	−0.330	0.035	0.852	0.719 (0.022, 23.324)
	Employed	3.388	4.260	0.039	29.621 (1.186, 739.708)
	The score of MOSE	0.693	12.560	<0.001	1.999 (1.363, 2.931)
	The score of Housebound Scale	−0.603	2.966	0.085	0.547 (0.276, 1.087)

NIHSS, National Institute of Health Stroke Scale; OR, odds ratio.

a The low-level of recovery poor class (Class 3) was used as the reference group for the dependent variable of the multinomial logistic regression.

Class 1, medium-level recovery good class; Class 2, high-level recovery flat class.

## 4 Discussion

To our knowledge, this represents the first study to longitudinally explore the life space trajectories of Chinese stroke patients. The analysis revealed three distinct classes of life space trajectories: high-level of recovery flat class, medium-level of good recovery class and low-level of poor recovery class. Meanwhile, age, limb sensory impairment, employment status and environment were identified as significant predictors associated with trajectory classification. These findings contribute to the theoretical basis for medical professionals to monitor the critical time points during the recovery period of stroke patients, and facilitate early identification of patients at risk of sustained life space restriction.

A cohort study of 173 stroke patients was examined from T0 to T3, and this study found that the sample showed an overall decreasing and then increasing trend in life space from before the onset of stroke to 6 months post-discharge. Among them, the life space level reached its nadir at 1 month post-discharge, followed by a gradual increase until stabilizing at 3 months post-discharge. The results of repeated measures ANOVA demonstrated that the scores of total life space and five levels were significantly lower than the other three time points in the first month post-discharge. These findings are consistent with previous prospective studies (40, 41). Unlike the higher-levels of life space (e.g., level 4), which demonstrate a gradual recovery trend, the lower-levels of life space (levels 1 and 2) display limited variation across various stages. Specifically, they decreased significantly at T1, with no sustained changes observed in other time intervals. This finding indicates that changes in life space are not uniform across hierarchical structures. Regarding the phenomenon that the changes in lower-level life space are not obvious, we hypothesize that the possible reason is that levels 1 and 2 reflect movement within the home environment (such as rooms, courtyards). For the vast majority of stroke patients, minimal physical effort is required to access these two types of areas, allowing the scores of levels 1 and 2 to remain consistently high and stable. Critically, this study suggested that stroke, a traumatic stress event, is associated with limitations in physical activities. The observed life space contraction may also be influenced by psychosocial factors such as kinesiophobia or reduced social participation (42, 43). The steep decline in life space at 1 month post-discharge suggests that this period may be particularly vulnerable for patients, potentially reflecting concurrent challenges in physical, psychological, and social domains that warrant comprehensive clinical attention. Healthcare professionals should reinforce discharge education, with particular emphasis on functional recovery and psychological adaptation in stroke patients, to mitigate the adverse effects of the disease on patients’ physiological, psychological, and social functioning. In addition, there was no significant difference between the total life space scores at T3 and T2. It showed that the increasing trend of life space in stroke patients began to slow down from 3 months post-discharge, which is consistent with the idea that there are growth and stabilization periods in life space restoration, as proposed by a Japanese scholar (44). Moreover, comparison of the scores of T0 to T3 of five levels of life space revealed that the score at T3 was significantly higher than that at T2 (*p* < 0.05) only in the level 4 (to town) of life space. This phenomenon may be attributed to the proximity of the first three levels to residential areas, representing routine daily activities, whereas level 5 (beyond town) exceeds typical mobility ranges for most patients during recovery. Level 4 mobility necessitates moderate physical capability and active engagement from patients. Therefore, level 4 scores may serve as a sensitive indicator of functional recovery in stroke patients.

In this study, 8% of the patients were classified into the high-level of recovery flat class (Class 2). The overall level of life space was high and exhibited steady progression, with patients experiencing minimal adverse effects from stroke onset. It is important to note that while the point estimate suggested a stable course at a high level, the associated statistical uncertainty was substantial. This is a common challenge when modeling small subgroups (*n* = 13, 8% of the sample), and the true trajectory may vary. Therefore, the most robust conclusion for this class is that they maintained a high level of life space throughout the study, while the exact pattern of change over time requires verification in larger samples. The multinomial logistic regression analysis identified employed status and positive environmental experiences as significant predictors for belonging to Class 2. A possible explanation for this association is that employed patients in this cohort were predominantly young to middle-aged males with substantial familial and societal responsibilities, often necessitating a prompt return to work. Furthermore, constrained sick leave durations were commonly associated with an early return to work after a brief recuperation. Although this class does not necessitate targeted interventions, it is essential to remind the patients to prioritize adequate rest, adhere to scheduled follow-up visits, and comply with prescribed medication regimens to prevent stroke recurrence. Additionally, the results of the multinomial logistic regression identified that a higher environmental score was a predictor of favorable life space trajectory. This finding underscores the importance of travel convenience and the availability of community assistive infrastructure, particularly for elderly patients with physical impairments, in relation to the feasibility to engage in outdoor mobility. Previous research corroborates this finding, demonstrating that favorable living environments and accessible community infrastructure are linked to greater exercise engagement and social participation among stroke patients (45). This highlights the need for healthcare professionals to assess the environmental context of stroke patients and advise on potential home adaptations. For instance, incorporating assistive devices and equipment can enhance the home environment, thereby improving patients’ perceived safety and travel convenience.

72% patients belonged to the medium-level of recovery good class (Class 1). The life space of patients in this class returned to pre-morbid levels by 3 months post-discharge and exceeded pre-morbid levels at 6 months post-discharge. This suggested that patients in this class achieved favorable recovery outcomes, with enhanced motor awareness compared to before. This may reflect the positive impact of stroke on patients, as they become aware of the importance of health management and adopt proactive coping strategies, such as improving lifestyle habits and engaging in physical exercise, to facilitate recovery. The results indicated that the absence of limb sensory deficit was the predictor of Class 1. Preserved sensory function has been linked in previous studies to reduced muscle tone, enhanced motor function, and improved limb mobility (46), which could facilitate greater life space recovery. Conversely, sensory dysfunction predisposes patients to balance impairments and restricted mobility, correlating with progressive decline in life space (47). In the past, healthcare professionals have prioritized the assessment and intervention of motor impairments in stroke survivors, frequently overlooking the diagnosis and rehabilitation of sensory dysfunction (48). This study demonstrated the critical role of sensory function in stroke rehabilitation, highlighting the need for medical personnel to assess and treat sensory impairments in stroke patients. And medical personnel should collaborate with rehabilitation teams to address and mitigate sensory dysfunction, thereby optimizing rehabilitation outcomes for stroke patients.

Finally, 20% of the patients were classified into the low-level of recovery poor class (Class 3). Post-discharge life space in this class remained below pre-stroke levels, indicating significant adverse effects of stroke and limited recovery capacity among these patients. Additionally, life space in this class contracted to its nadir at 1 month post-discharge, exhibited a statistically significant recovery by 3 months, but subsequently failed to maintain this momentum, demonstrating a declining trend by 6 months. This pattern strongly suggests that for this most vulnerable patient group, medium-term recovery may be transient and unstable. The subtle decline from T2 to T3 may indicate that patients enter a “rehabilitation plateau” or experience “rehabilitation burnout,” where the momentum and effectiveness of early rehabilitation wane without additional, sustained support. Consequently, the patients in the Class 3 represent a critical target for clinical intervention. Baseline analysis revealed that patients in this class were significantly more likely to be ≥60 years old. This association might be explained by age-related declines in muscle reserve, exercise tolerance, and balance function, compounded by slower disease recovery rates in elderly patients compared to younger and middle-aged individuals (49, 50). The combined impact of physiological and pathological factors is linked to reduced mobility and diminished activity engagement among elderly stroke patients post-discharge, accompanying progressive decline in life space. The multinomial logistic regression analysis indicated that healthcare professionals should prioritize elderly patients presenting with limb sensory deficits and suboptimal environmental experiences. These patients are at higher risk of belonging to Class 3 and experiencing the most severe and prolonged adverse outcomes. Therefore, proactive interventions by healthcare providers are essential, with a focus on mitigating physical sensory deficits and enhancing the patient’s home environment experience.

This study has several limitations. Firstly, the sample predominantly comprised mild-to-moderate stroke patients, as severely ill patients were unable to tolerate prolonged investigations during the T0, resulting in under representation and potential bias. Secondly, predictor variables were measured only at baseline without follow-up, limiting our ability to assess how changes in these variables over the recovery period might be associated with life space trajectories. Thirdly, the sample size of the high-level recovery flat class (*n* = 13, 8%) was relatively small. This may have obscured underlying heterogeneity within this group and reduced the power to identify its unique predictors.

## 5 Implications for nursing practice and research

The findings highlight the critical importance of implementing stage-specific rehabilitation plans aligned with patients’ recovery trajectories. The significant decline in life space at 1 month post-discharge underscores the need for early and intensive nursing interventions focused on functional recovery and psychological support during this vulnerable period. For patients in the low-level of recovery poor class—typically older adults with sensory impairment and negative environmental perceptions—nurses should prioritize sensory rehabilitation and environmental modifications (e.g., home safety adaptations, assistive devices) to enhance mobility safety and confidence. Additionally, employing the identified predictors may allow early identification of high-risk patients for tailored interventions. Future studies should focus on developing targeted interventions, particularly those addressing sensory function and environmental optimization, for high-risk subgroups. Future longitudinal studies with more frequent timepoints are warranted to further elucidate critical recovery periods and causal mechanisms.

## 6 Conclusion

The overall trend of life space was first decreasing and then increasing, with the level of life space falling to its lowest within 1 month of discharge, indicating that stroke is associated with significant damage to patients’ physical, psychological, and social functions in the short term. Notably, there are three distinct trajectories of life space among stroke patients, and patients in different trajectories profiles differed mainly in place of age, employment status, sensory impairment and environmental experience. The above predictors of trajectories provide the basis for future research to develop targeted interventions to improve life space, which may in turn support broader rehabilitation goals for stroke patients.

## Data Availability

The raw data supporting the conclusions of this article will be made available by the authors, without undue reservation.
