# Supplementary material for: The heterogeneous life space trajectories and predictors in stroke patients: a cohort study
Source: Front Neurol. 2025 Oct 3;16:1627893. doi: 10.3389/fneur.2025.1627893 (PMC12531049; doi:10.3389/fneur.2025.1627893)
Supplement: Supplementary file 1 [file Table_S1-S3.docx]

Supplementary Material

# Supplementary Tables

**TABLE S1** Baseline characteristics of the study population.

| Characteristics | n (%) | | | *P*^a^ | *P*^b^ |
| --- | --- | --- | --- | --- | --- |
|  | Overall (n=210) | Completed 3 follow-ups (n=173) | Lost to follow-up (n=37) |  |  |
| Age (years) |  |  |  | 0.965 | 0.889 |
| ＜60 | 83 (39.5) | 68 (39.3) | 15 (40.5) |  |  |
| ≥60 | 127 (60.5) | 105 (60.7) | 22 (59.5) |  |  |
| Sex |  |  |  | 0.559 | 0.076 |
| Male | 150 (71.4) | 128 (74.0) | 22 (59.5) |  |  |
| Female | 60 (28.6) | 45 (26.0) | 15 (40.5) |  |  |
| BMI (kg/m^2^) |  |  |  | 0.897 | 0.365 |
| ＜18.5 | 5 (2.4) | 3 (1.7) | 2 (5.4) |  |  |
| 18.5~23.9 | 78 (37.1) | 66 (38.2) | 12 (32.4) |  |  |
| ≥24 | 127 (60.5) | 104 (60.1) | 23 (62.2) |  |  |
| Ethnicity |  |  |  | 1.000 | 1.000 |
| Han | 208 (99.0) | 171 (98.8) | 37 (100) |  |  |
| Minority | 2 (1.0) | 2 (1.2) | 0 (0) |  |  |
| Religion |  |  |  | 0.624 | 0.223 |
| No | 190 (90.5) | 159 (91.9) | 31 (83.8) |  |  |
| Yes | 20 (9.5) | 14 (8.1) | 6 (16.2) |  |  |
| Marital status |  |  |  |  |  |
| Single | 9 (4.3) | 6 (3.5) | 3 (8.1) | 0.974 | 0.589 |
| Married | 179 (85.2) | 149 (86.1) | 30 (81.1) |  |  |
| Divorced | 9 (4.3) | 8 (4.6) | 1 (2.7) |  |  |
| widowed | 13 (6.2) | 10 (5.8) | 3 (8.1) |  |  |
| Educational attainment |  |  |  | 0.962 | 0.377 |
| Primary school or below | 28 (13.3) | 21 (12.1) | 7 (18.9) |  |  |
| Secondary school | 85 (40.5) | 68 (39.3) | 17 (45.9) |  |  |
| High school | 54 (25.7) | 48 (27.7) | 6 (16.2) |  |  |
| University or above | 43 (20.5) | 36 (20.8) | 7 (18.9) |  |  |
| Place of Residence |  |  |  | 0.695 | 0.146 |
| Urban | 165 (78.6) | 136 (78.6) | 29 (78.4) |  |  |
| Town | 43 (20.5) | 37 (21.4) | 6 (16.2) |  |  |
| Rural | 2 (0.9) | 0 (0) | 2 (5.4) |  |  |
| Living status |  |  |  | 0.954 | 0.573 |
| Alone | 35 (16.7) | 27 (15.6) | 8 (21.6) |  |  |
| With family | 175 (83.3) | 146 (84.4) | 29 (78.4) |  |  |
| Vocation |  |  |  | 1.000 | 0.615 |
| Farmer | 1 (0.5) | 1 (0.6) | 0 (0) |  |  |
| Functionary | 2 (1.0) | 1 (0.6) | 1 (2.7) |  |  |
| Teacher/Staff/Technician | 44 (21.0) | 38 (22.0) | 6 (16.2) |  |  |
| Self-employed laborer | 5 (2.4) | 4 (2.3) | 1 (2.7) |  |  |
| Retirement | 121 (57.6) | 98 (56.6) | 23 (62.2) |  |  |
| Liberal professions | 37 (17.6) | 31 (17.9) | 6 (16.2) |  |  |
| Employment status |  |  |  | 0.997 | 0.917 |
| Employed | 19 (9.0) | 15 (8.7) | 4 (10.8) |  |  |
| Employed (be on sick leave) | 49 (23.3) | 41 (23.7) | 8 (21.6) |  |  |
| Unemployed | 22 (10.5) | 19 (11.0) | 3 (8.1) |  |  |
| Retired | 120 (57.1) | 98 (56.6) | 22 (59.5) |  |  |
| Family income, thousand/m (RMB) |  |  |  | 0.946 | 0.380 |
| 1000-3000 | 2 (1.0) | 1 (0.6) | 1 (2.7) |  |  |
| 3001-5000 | 31 (14.8) | 27 (15.6) | 4 (10.8) |  |  |
| 5001-10000 | 116 (55.2) | 98 (56.6) | 18 (48.6) |  |  |
| ＞10000 | 61 (29.0) | 47 (27.2) | 14 (37.8) |  |  |
| Methods of payment of medical expenses |  |  |  | 0.957 | 0.432 |
| Self funded | 5 (2.4) | 0 (0) | 2 (5.4) |  |  |
| Employee medical insurance | 38 (18.1) | 33 (19.1) | 5 (13.5) |  |  |
| Urban medical insurance | 123 (58.6) | 99 (57.2) | 24 (64.9) |  |  |
| Rural cooperative medical service | 44 (21.0) | 41 (23.7) | 6 (16.2) |  |  |
| Type of stroke |  |  |  | 0.689 | 0.321 |
| Ischemic stroke | 193 (91.9) | 157 (90.8) | 36 (97.3) |  |  |
| Hemorrhagic stroke | 17 (8.1) | 16 (9.2) | 1 (2.7) |  |  |
| No. of stroke occurrences |  |  |  | 0.934 | 0.481 |
| 1 | 155 (73.8) | 129 (74.6) | 26 (70.3) |  |  |
| 2 | 44 (21.0) | 34 (19.7) | 10 (27.0) |  |  |
| ≥3 | 11 (5.2) | 10 (5.8) | 1 (2.7) |  |  |
| No. of other chronic diseases |  |  |  | 0.980 | 0.804 |
| 0 | 41 (19.5) | 35 (20.2) | 6 (16.2) |  |  |
| 1~2 | 144 (68.6) | 117 (67.6) | 27 (73.0) |  |  |
| ≥3 | 25 (11.9) | 21 (12.1) | 4 (10.8) |  |  |
| No. of functional impairments |  |  |  | 0.924 | 0.429 |
| 0 | 38 (18.1) | 34 (19.7) | 4 (10.8) |  |  |
| 1~2 | 127 (60.5) | 102 (59.0) | 25 (67.6) |  |  |
| ≥3 | 45 (21.4) | 37 (21.4) | 8 (21.6) |  |  |
| Whether to participate in social activities |  |  |  | 0.693 | 0.205 |
| Yes | 105 (50) | 83 (48.0) | 22 (59.5) |  |  |
| No | 105 (50) | 90 (52.0) | 15 (40.5) |  |  |

No, Number.

^a^Comparison of characteristics between a total of 210 participants and 173 participants who completed 3 follow-ups.

^b^Comparison of characteristics between173 participants who completed 3 follow-ups and 37 participants who were lost to follow-up.

**TABLE S2** Estimation results of unconditional nonlinear growth model (n=173).

| Model | Coefficient | | | Variation | | |
| --- | --- | --- | --- | --- | --- | --- |
|  | Intercept | Slope | Curve slope | Intercept | Slope | Curve slope |
| Unconditional nonlinear model | 71.331^a^ | -8.333^a^ | 2.854^a^ | 396.705^a^ | 359.370^a^ | 10.925 |

^a^*P* <0.01.

**TABLE S3** Univariate analysis of life space trajectories.

| Variables | Class 1 (n=125) | Class 2 (n=13) | Class 3 (n=35) | *F*/*H*/*χ^2^* | *P* |
| --- | --- | --- | --- | --- | --- |
| Age (years), n (%) |  |  |  | 15.193^a^ | 0.001 |
| ＜60 | 52 (41.6) | 10 (76.9) | 6 (17.1) |  |  |
| ≥60 | 73 (58.4) | 3 (23.1) | 29 (82.9) |  |  |
| Sex, n (%) |  |  |  | 7.941^a^ | 0.019 |
| Male | 96 (76.8) | 12 (92.3) | 20 (57.1) |  |  |
| Female | 29 (23.2) | 1 (7.7) | 15 (42.9) |  |  |
| NIHSS score on admission, n (%) |  |  |  | 6.065^a^ | 0.048 |
| Mild (2≤NIHSS≤4) | 92 (73.6) | 9 (69.2) | 18 (51.4) |  |  |
| Moderate and severe (NIHSS≥5) | 33 (26.4) | 4 (30.8) | 17 (48.6) |  |  |
| BMI (kg/m^2^), n (%) |  |  |  | 6.758^b^ | 0.100 |
| ＜18.5 | 2 (1.6) | 0 (0) | 2 (5.7) |  |  |
| 18.5~23.9 | 112 (89.6) | 11 (84.6) | 33 (94.3) |  |  |
| ≥24 | 11 (8.8) | 2 (15.4) | 0 (0) |  |  |
| Ethnicity, n (%) |  |  |  | 3.901^b^ | 0.185 |
| Han | 124 (99.2) | 12 (92.3) | 35 (100) |  |  |
| Minority | 1 (0.8) | 1 (7.7) | 0 (0) |  |  |
| Religion, n (%) |  |  |  | 1.205^b^ | 0.565 |
| No | 115 (92.0) | 13 (100) | 31 (88.6) |  |  |
| Yes | 10 (8.0) | 0 (0) | 4 (11.4) |  |  |
| Marital status, n (%) |  |  |  | 7.501^b^ | 0.181 |
| Single | 6 (4.8) | 0 (0) | 0 (0) |  |  |
| Married | 109 (87.2) | 12 (92.3) | 28 (80.0) |  |  |
| Divorced | 3 (2.4) | 1 (7.7) | 4 (11.4) |  |  |
| widowed | 7 (5.6) | 0 (0) | 3 (8.6) |  |  |
| Educational attainment, n (%) |  |  |  | 10.908^b^ | 0.074 |
| Primary school or below | 14 (11.2) | 1 (7.7) | 6 (17.1) |  |  |
| Secondary school | 50 (40.0) | 3 (23.1) | 15 (42.9) |  |  |
| High school | 40 (32.0) | 2 (15.4) | 6 (17.1) |  |  |
| University or above | 21 (16.8) | 7 (53.8) | 8 (22.9) |  |  |
| Place of Residence, n (%) |  |  |  | 2.062^a^ | 0.834 |
| Urban | 96 (76.8) | 11 (84.6) | 29 (82.9) |  |  |
| Rural or town | 29 (23.3) | 2 (15.4) | 6 (17.1) |  |  |
| Employment status, n (%) |  |  |  | 25.350^a^ | ＜0.001 |
| Employed | 42 (33.6) | 11 (84.6) | 3 (8.6) |  |  |
| Unemployed | 83 (66.4) | 2 (15.4) | 32 (91.4) |  |  |
| Family income, thousand/m (RMB), n (%) |  |  |  | 8.229^b^ | 0.072 |
| ≤5000 | 21 (16.8) | 0 (0) | 7 (20.0) |  |  |
| 5001-10000 | 74 (59.2) | 5 (38.5) | 19 (54.3) |  |  |
| ＞10000 | 30 (24.0) | 8 (61.5) | 9 (25.7) |  |  |
| Methods of payment of medical expenses, n (%) |  |  |  | 3.521^a^ | 0.172 |
| Medical insurance | 91 (72.8) | 12 (92.3) | 29 (82.9) |  |  |
| Rural cooperative medical service | 34 (27.2) | 1 (7.7) | 6 (17.1) |  |  |
| Type of stroke, n (%) |  |  |  | 3.397^b^ | 0.130 |
| Ischemic stroke | 115 (92.0) | 13 (100) | 29 (82.9) |  |  |
| Hemorrhagic stroke | 10 (8.0) | 0 (0) | 6 (17.1) |  |  |
| Whether first stroke, n (%) |  |  |  | 7.263^a^ | 0.026 |
| Yes | 98 (78.4) | 11 (84.6) | 20 (57.1) |  |  |
| No | 27 (21.6) | 2 (15.4) | 15 (42.9) |  |  |
| No. of stroke occurrences, n (%) |  |  |  | 8.049^b^ | 0.064 |
| 1 | 98 (78.4) | 11 (84.6) | 20 (57.1) |  |  |
| 2 | 22 (72.6) | 2 (15.4) | 10 (28.6) |  |  |
| ≥3 | 5 (4.0) | 0 (0) | 5 (14.3) |  |  |
| No. of other chronic diseases, n (%) |  |  |  | 5.440^a^ | 0.220 |
| 0 | 24 (19.2) | 5 (38.5) | 6 (17.1) |  |  |
| 1~2 | 87 (69.6) | 8 (61.5) | 22 (62.9) |  |  |
| ≥3 | 14 (11.2) | 0 (0) | 7 (20.0) |  |  |
| No. of functional impairments, n (%) |  |  |  | 0.555^a^ | 0.758 |
| ＜3 | 100 (80.0) | 10 (76.9) | 26 (74.3) |  |  |
| ≥3 | 25 (20.0) | 3 (23.1) | 9 (25.7) |  |  |
| Presence of limb movement disorder, n (%) |  |  |  | 7.644^a^ | 0.022 |
| Yes | 74 (59.2) | 4 (30.8) | 26 (74.3) |  |  |
| No | 51 (40.8) | 9 (69.2) | 9 (25.7) |  |  |
| Presence of limb sensory impairment, n (%) |  |  |  | 7.595^a^ | 0.022 |
| Yes | 49 (39.2) | 8 (61.5) | 22 (62.9) |  |  |
| No | 76 (60.8) | 5 (38.5) | 13 (37.1) |  |  |
| Presence of facial paralysis, n (%) |  |  |  | 1.548^b^ | 0.564 |
| Yes | 17 (13.6) | 0 (0) | 4 (11.4) |  |  |
| No | 108 (86.4) | 13 (100) | 31 (88.6) |  |  |
| Presence of visual field defect, n (%) |  |  |  | 0.553^b^ | 0.798 |
| Yes | 5 (4.0) | 0 (0) | 2 (5.7) |  |  |
| No | 120 (96.0) | 13 (100) | 33 (94.3) |  |  |
| Presence of visual impairment, n (%) |  |  |  | 1.364^b^ | 0.644 |
| Yes | 12 (9.6) | 2 (15.4) | 5 (14.3) |  |  |
| No | 113 (90.4) | 11 (84.6) | 30 (85.7) |  |  |
| Presence of dysphagia, n (%) |  |  |  | 0.396^b^ | 1.000 |
| Yes | 8 (6.4) | 1 (7.7) | 2 (5.7) |  |  |
| No | 117 (93.6) | 12 (92.3) | 33 (94.3) |  |  |
| Presence of ataxia, n (%) |  |  |  | 0.977^b^ | 0.638 |
| Yes | 9 (7.2) | 1 (7.7) | 4 (11.4) |  |  |
| No | 116 (92.8) | 12 (92.3) | 31 (88.6) |  |  |
| Presence of headaches or dizziness, n (%) |  |  |  | 0.652^a^ | 0.722 |
| Yes | 18 (14.4) | 2 (15.4) | 7 (20.0) |  |  |
| No | 107 (85.6) | 11 (84.6) | 28 (80.0) |  |  |
| Whether to participate in social life, n (%) |  |  |  | 1.204^a^ | 0.548 |
| Yes | 62 (49.6) | 7 (53.8) | 14 (40.0) |  |  |
| No | 63 (50.4) | 6 (46.2) | 21 (60.0) |  |  |
| Neurological deficit, n (%) |  |  |  | 8.505^a^ | 0.014 |
| Mild | 110 (88.0) | 12 (92.3) | 24 (68.6) |  |  |
| Moderate and severe | 15 (12.0) | 1 (7.7) | 11 (31.4) |  |  |
| Self-efficacy, n (%) |  |  |  | 1.168^a^ | 0.558 |
| Low level | 112 (89.6) | 11 (84.6) | 33 (94.3) |  |  |
| Medium level | 13 (10.4) | 2 (15.4) | 2 (5.7) |  |  |
| Hope, n (%) |  |  |  | 7.651^a^ | 0.087 |
| Lower-middle level | 53 (42.4) | 5 (38.5) | 20 (57.1) |  |  |
| High level | 72 (57.6) | 8 (61.5) | 15 (42.9) |  |  |
| Psychological distress, n (%) |  |  |  | 7.926^a^ | 0.079 |
| No distress | 83 (66.4) | 9 (69.2) | 16 (45.7) |  |  |
| Mild | 31 (24.8) | 3 (23.1) | 10 (28.6) |  |  |
| Moderate and severe | 11 (8.8) | 1 (7.7) | 9 (25.7) |  |  |
| Social support, n (%) |  |  |  | 6.758^b^ | 0.100 |
| Low level | 2 (1.6) | 0 (0) | 2 (5.7) |  |  |
| Medium level | 112 (89.6) | 11 (84.6) | 33 (94.3) |  |  |
| High level | 11 (8.8) | 2 (15.4) | 0 (0) |  |  |
| The score of Housebound Scale, Mean±SD | 6.15±1.44 | 5.77±1.24 | 6.97±2.22 | 4.248^c^ | 0.016 |
| Economic status, M (P_25_, P_75_) | 31 (26, 34) | 36 (27, 37.5) | 32 (20, 36) | 5.294^d^ | 0.071 |
| The score of MOSE, M (P_25_, P_75_) | 27 (25, 29) | 30 (29, 31) | 22 (14, 25) | 41.330^d^ | ＜0.001 |

NIHSS, National Institute of Health Stroke Scale; M, median.

^a^χ^2^ test.

^b^Fisher's exact test.

^c^ANOVA.

^d^Nonparametric tests.
